# Development of a Classification Model for Intramammary Infection at Dry-Off Using Indirect Cow-Level Indicators

**DOI:** 10.3390/ani16172719

**Published:** 2026-09-01

**Authors:** Luca Barin, Giorgio Marchesini, Andrea Simonetto, Katia Capello, Federico Martignago, Lara Biasio, Erica Littamè, Lara Tasinato, Antonio Barberio

**Affiliations:** 1 Department of Animal Medicine, Production and Health, University of Padua, Viale dell’Università 16, 35020 Legnaro, Italy; giorgio.marchesini@unipd.it; 2 Istituto Zooprofilattico Sperimentale delle Venezie, Viale dell’Università 10, 35020 Legnaro, Italy; asimonetto@izsvenezie.it (A.S.); kcapello@izsvenezie.it (K.C.); fmartignago@izsvenezie.it (F.M.); lbiasio@izsvenezie.it (L.B.); elittame@izsvenezie.it (E.L.); ltasinato@izsvenezie.it (L.T.); abarberio@izsvenezie.it (A.B.)

**Keywords:** selective dry cow therapy, antimicrobial reduction, classification model, multivariable logistic regression model

## Abstract

Reducing antimicrobial use in dairy cattle requires practical methods to identify cows that need antimicrobial treatment at dry-off. The objective of this study was to develop and evaluate a classification model using on-farm indirect indicators as a first step toward identifying cows with intramammary infection (IMI) at dry-off. The study was conducted on a single commercial dairy farm and included 301 cows. Quarter milk samples were collected before dry-off and used as the reference method to define IMI. Cow-level predictors included somatic cell count (SCC) history, clinical mastitis history, parity, teat health score, and genetic values for SCC, which were combined in a multivariable logistic regression model internally validated using a 75/25 training-testing split. Overall, 1190 quarters were examined, and 129 cows were classified as infected with at least one major mammary pathogen at dry-off. The best-performing model included genetic value for SCC, linear score at selected test days, and clinical mastitis history, reaching an accuracy of 0.75 (0.64–0.84), a sensitivity of 0.80 (0.63–0.92), a specificity of 0.71 (0.55–0.84), and an AUC of 0.76 (0.65–0.87). These results suggest that combining indirect cow-level indicators may support treatment allocation at dry-off, although external validation across multiple herds is required.

## Simple Summary

Reducing unnecessary antibiotic use in dairy cows is important, but cows that are infected at dry-off still need appropriate treatment to preserve udder health. Selective dry cow therapy is based on treating only cows that are likely to have an udder infection at the end of lactation, but this requires a reliable way to identify infected cows before treatment. In this study, milk samples collected at dry-off were used to determine which cows were infected, and routinely available farm data were tested as possible predictors. These included milk quality records, clinical mastitis history, milk production, genetic information, parity, and teat end condition. The best model included genetic value for somatic cell count, test-day somatic cell information and clinical mastitis history. These findings suggest that combining data already available on farms may help improve treatment decisions at dry-off, reducing unnecessary antimicrobial use and improving animal welfare.

## 1. Introduction

Reducing antimicrobial use in food-producing animals is a major priority for public health, animal production and consumer confidence because antimicrobial exposure can contribute to the selection of antimicrobial resistance [1,2,3]. In dairy cattle, a substantial proportion of antimicrobial use in adult cows is associated with mastitis treatment and prevention, especially for blanket dry cow therapy (BDCT), where all quarters of all cows are treated at dry-off [4,5,6]. BDCT was widely adopted since the 1960s to cure existing intramammary infection (IMI) present at dry-off and to prevent the development of new infections during the dry period, but the need to reduce unnecessary antimicrobial use has recently supported the transition toward selective dry cow therapy [7,8,9].

Selective dry cow therapy (SDCT) aims to reduce antimicrobial use while maintaining udder health by treating only cows considered likely to have an IMI, and this approach may also provide economic benefits by avoiding unnecessary antimicrobial treatments [10,11,12]. However, the effectiveness of SDCT depends strongly on the ability to identify cows that require antimicrobial treatment at dry-off [13,14,15].

Different approaches have been used to identify cows with IMI at dry-off, including laboratory or on-farm bacteriological culture of quarter milk samples [16,17], cow-side tests such as the California Mastitis Test [18], and algorithms based on routinely recorded data such as somatic cell count (SCC) and clinical mastitis history [19,20]. Bacteriological culture provides direct information on infection status and pathogen group, and by identifying the causative pathogen it can also reveal whether an infection, although present, should be treated or not with antimicrobials; however, it requires aseptic sampling, laboratory or on-farm culture capacity, and a variable amount of time before the results are available. It can also be expensive [16,17,21]. Cow-side tests and record-based algorithms are faster, cheaper and easier to implement, but represent an indirect method of classification and may therefore misclassify infected or noninfected animals [21,22,23].

Since several indirect indicators are usually available from routine farm records, including lactation data, SCC history, clinical mastitis history, genetic information and parity, their combined use may provide a practical approach to classify cows according to their probability of IMI at dry-off. However, the predictive value of combining these routinely available cow-level indicators to support treatment allocation at dry-off remains insufficiently defined.

The aim of this study was to develop a predictive classification model to identify cows with IMI at dry-off and to support treatment allocation using indirect cow-level indicators available on-farm before dry-off.

## 2. Materials and Methods

### 2.1. Study Overview

To address the study objective, quarter milk samples collected at dry-off were used as the reference method to define IMI status, and routinely available cow-level information was evaluated as candidate predictors. Cow-level data from the current lactation, including milk production, SCC records, clinical mastitis history, genetic information, and parity, were obtained from farm records and Dairy Herd Improvement (DHI) data. Teat-end condition was also evaluated at dry-off and included among the candidate predictors. These data were used to develop and select a predictive model for IMI at dry-off based on statistical fit and predictive performance.

### 2.2. Farm Description and Animal Selection

The study was conducted from November 2023 to August 2024 on a commercial dairy farm in the Veneto region of Italy with an average herd size of 540 lactating cows. Cows were milked in a rotary milking parlor and housed in cubicle barns with straw during lactation. They were fed a corn-based total mixed ration and had access to water ad libitum. Cows were dried off approximately 60 days before their expected calving date. All cows dried off during the study period were sampled when they met the inclusion criteria, regardless of parity. Cows were eligible for inclusion if DHI records were available for the entire lactation and if the last DHI test had been performed within 30 days before dry-off. The final dataset included 301 cows, of which 110 were primiparous, 87 secondiparous and 104 were multiparous.

### 2.3. Milk Samples, Bacteriological Analysis and Teat Score Collection

Quarter milk samples were collected by trained personnel from 301 cows between 7 and 0 days before dry-off following aseptic procedures [24]. After collection, samples were frozen at −20 °C until bacteriological examination, for up to 4 weeks. Although freezing may affect the recovery of some milk-borne pathogens, its impact is pathogen-dependent, and short-term frozen storage does not necessarily cause a substantial loss of pathogen viability [25,26]. Before the analysis, samples were thawed and then cultured according to National Mastitis Council guidelines [27,28]. Briefly, 0.01 mL of each quarter sample was spread onto esculin blood agar, incubated aerobically at 37 °C and examined after 24 and 48 h. A quarter was considered positive for a major pathogen when at least one colony was recovered, corresponding to the detection limit of 100 CFU/mL for the volume plated. Isolates were identified by matrix-assisted laser desorption/ionization time-of-flight mass spectrometry using the MALDI Biotyper system (Bruker Daltonics GmbH, Bremen, Germany), and classified as major or minor mammary pathogens as shown by Lipkens et al. [29]. Major pathogens included *Streptococcus* and *streptococcus*-like organisms, *S. aureus*, *S. agalactiae*, *Enterococcus* spp., *Pseudomonas* spp., *Serratia liquefaciens*, *Escherichia coli*, *Trueperella pyogenes*, and other Gram-positive bacteria. Samples with coinfection by 3 or more etiological agents were considered contaminated at collection and excluded from the usable dataset. Bacteriological analysis of quarter milk samples collected at dry-off was used as the reference method to define IMI status: a cow was considered infected when at least one quarter milk sample was positive for a major mammary pathogen. This cow-level definition aligns with the objective of SDCT, in which the presence of a major pathogen in even a single quarter warrants treatment of the whole cow; minor pathogens were not considered, as they do not typically justify antimicrobial treatment at dry-off [30].

Teat-end condition was evaluated in enrolled cows between 7 and 0 d before dry-off, at the same time as the milk sample collection and was assessed at the end of milking by a trained operator using the method described by Mein et al. [31], with a teat-end score ranging from 1 to 4.

### 2.4. Cow-Level Data Collection

Retrospective cow-level data included milk production at dry-off, SCC records, clinical mastitis events recorded during the current lactation, parity and genetic values for SCC. Individual cow data were obtained electronically from the information and the DHI records provided by the regional Breeders Association and uploaded into the farm management system Dairy Comp 305 (version 24.3; Valley Agricultural Software, Inc., Tulare, CA, USA). Only records referring to the current lactation and the dry-off period were used.

### 2.5. Data and Variable Creation

Productive, health, and genetic data were organized in Microsoft Excel for Microsoft 365 (Microsoft Corp., Redmond, WA, USA). The data collected were used to evaluate the association between selected cow-level indicators and IMI at dry-off. Candidate predictors included SCC records from the current lactation, clinical mastitis history during the current lactation, milk production before dry-off, teat-end condition assessed by teat score, parity and genetic values for somatic cell (gen_cell). Gen_cell is a genetic estimated breeding value expressing a cow’s inherited predisposition for SCC, derived from national genetic evaluations and expressed on a standardized index scale centered on a baseline of 100. Values above 100 indicate a genetic predisposition toward lower SCC and thus greater resistance to IMI, whereas values below 100 indicate a predisposition toward higher SCC and greater susceptibility. A summary of the variables used in the analysis is reported in Table 1 and descriptive statistics of continuous variables are reported in Table A1 and in Table A2 for categorical values.

SCC data were transformed to linear score (LS), which expresses SCC on a logarithmic scale. For each cow, SCC history was evaluated using all DHI records available from the current lactation. Because the number of DHI records differed among cows according to lactation length and dry-off date, test-day records were organized according to their proximity to dry-off, with LS_L1 corresponding to the last DHI test before dry-off, LS_L2 to the second-last test, and so on. Since all cows had at least six DHI records, the six test-days closest to dry-off (LS_L1 to LS_L6) were retained as candidate predictors, ensuring a complete and directly comparable SCC history for every animal with no missing values.

Clinical mastitis history (num_mast) was defined as a binary variable indicating the presence of at least one clinically diagnosed mastitis case during the current lactation, regardless of microbiological confirmation (0 = no cases; 1 = at least one case). Milk production on the day of dry-off was included as a continuous variable and was defined as milk_dry-off. Teat-end condition was transformed into a binary variable, with cows classified as having altered teat-end condition when at least one teat had a teat score > 2. Parity was categorized into three groups: first lactation, second lactation, and greater than second lactation.

The outcome of interest was IMI at dry-off, defined as the presence of at least one quarter milk sample positive for a major mammary pathogen. Before model development, data were examined to evaluate variable distributions, identify potential outliers, and assess the suitability of the selected indicators.

### 2.6. Model Development and Selection

Due to the binary nature of the outcome variable (presence/absence of IMI at dry-off), a multivariable logistic regression model (MLRM) was used to quantify the association between candidate cow-level predictors and the probability of IMI at dry-off. To evaluate predictive performance, the dataset was randomly split, stratified by IMI status, into a training set (75% of the observations) and a validation set (the remaining 25%). The training set was used for fitting the models, whereas the validation set was used to assess predictive ability. A total of 11 candidate variables, corresponding to 12 estimated parameters (parity being represented by two dummy variables), were considered before selection. No formal a priori sample size calculation with respect to the number of candidate covariates was performed; all cows meeting the inclusion criteria were included, and the implications of the available sample size are discussed in Section 4.

Predictors were selected through a forward stepwise procedure, and competing models were compared based on statistical adequacy criteria, including the Akaike information criterion and the quality of the residuals’ diagnostics. Although the known limitations of stepwise procedures are acknowledged, the combined use of forward selection with AIC-based model comparison and residual diagnostics represents an established approach in analogous studies, suitable for identifying parsimonious models from a limited set of biologically meaningful candidate predictors [32,33,34].

Pairwise correlations among candidate predictors were assessed in the training set, showing that the six LS_Lx variables were highly correlated with each other. Stepwise selection can be unstable in the presence of correlated predictors; however, such instability primarily affects the identification of which individual variable within a correlated group is retained, rather than the joint predictive contribution of the group as a whole [35]. As the primary objective of this study was predictive rather than interpretive, and since the validation set was obtained by random split from the same population, collinearity was not expected to compromise predictive performance.

The significance level was set at 0.05; however, for variable selection during the stepwise procedure a more liberal *p*-value threshold of 0.10 was adopted to explore whether the inclusion of additional predictors could improve predictive performance, since stricter criteria can fail to retain relevant predictors [36]. Multicollinearity among the predictors retained in the final model was assessed using variance inflation factors (VIF). Interactions between predictors were not considered.

For each candidate MLRM, the decision cut-off was tuned on the training set only, by pooling the out-of-fold probabilities from 10-fold cross-validation and selecting the threshold maximizing Youden’s index. The model was then refitted on the full training set and evaluated on the held-out validation set using this cut-off.

The predictive performance of the fitted MLRM was benchmarked against two rule-based algorithms adapted from the literature. The first (SCC1), adapted from Rowe et al. [19], classified cows as having an IMI when the SCC was ≥200,000 cells/mL in at least one of the DHI tests, or when more than one clinical mastitis case was recorded during the current lactation. The second (SCC3), adapted from Lipkens et al. [29], classified cows as infected when the SCC was ≥200,000 cells/mL in at least one of the last three DHI tests before dry-off.

Model performance on the test set was summarized using accuracy, balanced accuracy, sensitivity, specificity, positive and negative predictive values, Cohen’s kappa, the area under the ROC curve (AUC), and the Brier score. Differences in classification performance between the MLRM and the rule-based algorithms on the validation set were assessed using McNemar’s test, with *p*-values adjusted for multiple comparisons using the Holm correction.

All analyses were performed in R version 4.5.2 (R Foundation for Statistical Computing, Vienna, Austria). To ensure reproducibility, the random seed was set to 42 prior to data splitting and model fitting. The following packages were used: car (v 3.1-3), caret (v 7.0-1) and pROC (v 1.19.0.1).

## 3. Results

### 3.1. Bacteriological Analysis and Teat Score

A total of 1204 quarters was expected from 301 cows at dry-off; however, 14 cows had only 3 functional quarters, resulting in 1190 examined quarters. The categorization of quarters and animals is represented in Table 2. Overall, 1016 quarters (85%) were bacteriologically positive, whereas 152 quarters (13%) were negative and 22 quarters (2%) were classified as contaminated. Two-pathogen coinfection was detected in 154 quarters (13%). 241 quarters (20%) were positive for at least one major pathogen, and 129 cows (43%) were classified as having at least one quarter infected by a major pathogen at dry-off.

A summary of the pathogens isolated is reported in Table 3. Non-aureus staphylococci were the most frequently isolated bacteria, accounting for 844 isolates (72%). Among major pathogens, *S. uberis* was the most common isolate, detected in 159 quarters (14%), followed by *Pseudomonas* spp. in 27 quarters (2.3%), *Serratia liquefaciens* in 22 quarters (1.9%) and *Enterococcus* spp. in 21 (1.8%) quarters.

The distribution of teat health scores recorded is reported in Table 4. Score 1 and 2 accounted for 86% of the quarters evaluated. At the cow level, 73 cows (24%) had at least one teat with a score greater than 2.

### 3.2. Feature Selection and Modelling

The final MLRM retained LS_L1, LS_L3, num_mast, and gen_cell as predictors of IMI at dry-off (Table 5), yielding the following estimated equation:

$$logit\left(P\left(IMI=1\right)\right)=5.14+0.22\times LS_{L1}+0.33\times LS_{L3}+0.60\times num_{mast}-0.08\times gen_{cell}$$

Among these variables, LS_L3 was significantly associated with IMI at dry-off (*p* = 0.01), whereas LS_L1, num_mast, and gen_cell were retained in the model because they contributed to model fit and predictive performance, although their *p*-values were between 0.05 and 0.10. Variance inflation factors for the retained predictors ranged from 1.03 to 1.43 (LS_L1 = 1.37, LS_L3 = 1.43, num_mast = 1.03, gen_cell = 1.12), indicating negligible collinearity.

### 3.3. Model Performances

The performances of the MLRM are reported in Table 6, alongside two rule-based SDCT models reported in the literature [19,29]. The probability threshold selected by 10-fold cross-validation was 0.35. When applied to the validation dataset, the model correctly classified 57 out of 76 cows, corresponding to an overall accuracy and balanced accuracy of 0.75, with Cohen’s kappa of 0.50. Specifically, 7 of 35 infected cows were classified as not infected, whereas 12 of 41 noninfected cows were classified as infected (Table A3). The MLRM showed a higher point estimate of accuracy than both rule-based algorithms (SCC1: 0.645; SCC3: 0.658). Results of McNemar’s exact tests are reported in Table 7. The tests did not detect statistically significant differences between the MLRM and either benchmark (MLRM vs. SCC1: 14 vs. 6 discordant pairs in favor of the MLRM, *p* = 0.115; MLRM vs. SCC3: 13 vs. 6, *p* = 0.167; Holm-adjusted *p*-values 0.345 for both comparisons). The two rule-based algorithms performed similarly to each other (SCC1 vs. SCC3, *p* = 1.000). The direction of the discordant pairs consistently favored the MLRM over both rule-based classifiers, but the limited number of discordant observations on the validation set limited the ability of the test to reach statistical significance.

## 4. Discussion

In the present study, the prevalence of IMI at dry-off (43%) was higher than values commonly reported in the literature [19,29]. Non-aureus staphylococci were the most frequently isolated bacteria, whereas *S. uberis* was the predominant major pathogen. Because minor pathogens were excluded from the definition of the outcome, their presence may have influenced some indirect indicators without contributing to the classification of IMI, potentially reducing the apparent predictive value of these indicators. Nevertheless, this definition was chosen to reflect the treatment-oriented objective of the study, as minor pathogens do not generally justify antimicrobial treatment at dry-off. No *S. aureus* was isolated during the study period despite the relatively high prevalence of IMI. This may be related to the specific herd situation, as the farm had implemented a rigorous *S. aureus* control and eradication program and had remained free from detected *S. aureus* infection during the study period.

Only a limited number of variables were selected in the MLRM, which may improve the practical applicability of the classification system. The inclusion of genetic value for SCC in the best-performing model suggests that genetic information may provide additional value beyond routinely used phenotypic indicators. That is particularly relevant because genetic information is increasingly available in commercial dairy herds and could support a more individualized and integrated approach to udder health risk classification.

Clinical mastitis history was also retained as an important predictor; animals with at least one clinical mastitis case during the current lactation had a higher probability of IMI at dry-off. This finding supports the continued use of clinical mastitis history in treatment allocation protocols and the value of recording continuously this parameter on farm.

The selection of LS_L1 and LS_L3, but not LS_L2, may reflect overlap among consecutive SCC-derived indicators. DHI SCC tests were collected approximately every 35 days; therefore, consecutive LS records close to dry-off may represent udder health information collected within a relatively short interval. The information carried by LS_L2 may have been partially captured by LS_L1 and LS_L3, especially because udder health status can persist over time, consistently with the median recovery time for subclinical mastitis of 91 days reported by Kirkeby et al. [37], which may reduce the additional predictive contribution of intermediate LS measurements.

Altered teat-end condition may increase the risk of IMI, therefore teat health score was included among the candidate predictors [38]. In this herd, teat-end condition indicated a potential udder health risk because 24% of cows had at least one teat with a score of 3 or greater, whereas the remaining 76% of cows had all teats scored 2 or less, slightly below the 80% threshold considered desirable [31]. However, teat score was not retained in the final model, suggesting that teat-end condition contributed less to the prediction of IMI at dry-off than the other selected variables, in agreement with the findings of McDougall et al. [22].

Although milk production at dry-off has been considered a risk factor for new IMI during the dry period, this association is mainly related to milk leakage after dry-off [39,40]. In the present study, the outcome was IMI already present at dry-off; therefore, it is understandable that milk production at dry-off was not retained as a predictor in the final model.

The predictive model developed in this study showed higher point estimates for accuracy and sensitivity than the rule-based approaches used for comparison, although the confidence intervals overlapped [19,29]. The MLRM reached an accuracy and balanced accuracy of 0.75, with sensitivity of 0.80 and specificity of 0.71 suggesting that combining selected cow-level indicators may reduce misclassification and provide a more balanced approach to identifying cows with IMI at dry-off.

The main limitation of this study is that the model was developed and validated using data from a single farm with high IMI prevalence. Therefore, the results may reflect the specific prevalence, pathogen distribution, management practices, and recording quality of that herd. External validation on a larger number of farms with different management systems and udder health profiles is needed before the model can be generalized and implemented broadly in SDCT programs.

Beyond the single-farm setting, several additional limitations should be acknowledged. First, the sample size was limited, which constrains the statistical power of the analysis and the precision of the parameter estimates. This is particularly relevant when considered alongside the number of candidate predictor parameters evaluated during model development, as a high ratio of candidate predictors to available observations increases the risk of overfitting. Consequently, the model may capture patterns that are specific to this dataset rather than generalizable relationships, which could lead to an optimistic estimate of predictive performance when the model is applied to new data. Second, only cows with complete DHI recordings were included, which could in principle introduce a selection bias. In practice, however, in modern dairy farms it is very rare to find a cow without DHI records, so this is unlikely to bias the study population meaningfully; the more relevant constraint is the applicability of the model in herds that do not participate in DHI recording at all. A similar consideration applies to the genetic predictors used by the model. While not every farm currently has genetic data available, an increasing proportion of modern dairy farms do collect this information, so its availability is becoming less of a barrier over time. Nevertheless, in herds where DHI or genetic data are not routinely recorded or accessible, the practical applicability of the model may be reduced, further underscoring the need for external validation and, potentially, the development of alternative model versions based on more widely available predictors. The decision threshold was selected by maximizing Youden’s index and therefore did not explicitly incorporate different clinical or economic costs of false-negative and false-positive classifications. Future studies should evaluate cost-sensitive thresholds according to herd-specific treatment objectives.

Despite these limitations, the integration of genetic and production data already available on farm showed promising results and may support the future development of more accurate classification systems for treatment allocation at dry-off, reducing antimicrobial use while maintaining cow health and therefore preserving animal welfare.

## 5. Conclusions

The internally evaluated model showed potential for classifying major pathogen IMI at dry-off in the study herd. However, methodological refinement, assessment of calibration and clinical utility, and external validation across multiple herds are required before the model can be considered for treatment allocation.

## Figures and Tables

**Table 1:** Variables evaluated for model selection.

Variable	Type of Variable	Description
LS_L1-LS_L6 ^1^	Continuous	Logarithmic transformation of SCC value recorded at test day
milk_dry-off	Continuous	Milk production recorded at dry-off day
gen_cell	Continuous	Genetic value for SCC
lact	Categorical	Parity class: primiparous = 1, secondiparous = 2, multiparous = 3
num_mast	Binary	Occurrence of clinical mastitis during the current lactation (0 = no cases; 1 = ≥1 case)
teat_level	Binary	1 if at least one quarter had a teat score > 2, 0 otherwise

^1^ Linear score (LS) values were obtained from Dairy Herd Improvement tests. All cows had at least six test-day records in the current lactation, therefore the six records closest to dry-off were retained as candidate predictors.

**Table 2:** Number of quarters and animals infected.

Parameter	*n*	%
Cows infected by major pathogens	129	43
Total of cows	301 ^1^	-
Quarters positive ^2^	1016	85
Quarters with coinfection	154	13
Quarters positive with major pathogens	241	20
Quarters negative	152	13
Quarters contaminated	22	2
Total of quarters	1190 ^1^	-

^1^ A total of 1204 quarters was expected from 301 cows; however, 14 cows had only 3 functional quarters, resulting in 1190 examined quarters. ^2^ Quarters positive: quarters that were not contaminated or negative.

**Table 3:** Pathogens isolated during bacteriological analysis on a total of 1170 isolates.

Classification	Bacteria	*n* of Isolates (%)
Major pathogens	*Streptococcus uberis*	159 (14%)
	*Pseudomonas* spp.	27 (2.3%)
	*Serratia liquefaciens*	22 (1.9%)
	*Enterococcus* spp.	21 (1.8%)
	Other streptococci	13 (1.1%)
	*Trueperella pyogenes*	2 (0.2%)
	*Escherichia coli*	1 (0.1%)
Minor pathogens	Non-aureus staphylococci	844 (72%)
	*Corynebacterium* spp.	30 (2.6%)
	Other Enterobacteriaceae	8 (0.7%)
	Other pathogens	43 (3.7%)

**Table 4:** Distribution of teat health score at quarter and cow level.

Teat Score	Number of Quarters (%)	Number of Animals (%) ^1^
Score 1	769 (65%)	126 (42%)
Score 2	252 (21%)	102 (34%)
Score 3	124 (10%)	50 (17%)
Score 4	45 (4%)	23 (8%)

^1^ Cow-level distribution was based on the highest teat score recorded for each cow.

**Table 5:** Variables chosen for the multivariable logistic regression model following stepwise regression.

Coefficients ^1^	Estimate	Std. Error	OR (95% CI)	*p*-Value
(Intercept)	5.14	4.36	-	0.24
LS_L1	0.22	0.13	1.25 (0.97–1.62)	0.09
LS_L3	0.33	0.13	1.39 (1.08–1.8)	0.01
num_mast	0.60	0.36	1.82 (0.90–3.70)	0.09
gen_cell	−0.08	0.04	0.92 (0.85–1.01)	0.07

^1^ LS_L1 = Linear Score at the last Dairy Herd Improvement test before dry-off, LS_L3 = Linear Score at the third last Dairy Herd Improvement test before dry off, num_mast = occurrence of at least one clinical mastitis case, gen_cell = genetic value for somatic cell count.

**Table 6:** Performances in identifying major-pathogen intramammary infection at dry-off of the proposed model compared with two rule-based models adapted from the literature. All three models were evaluated on the same validation dataset from the study herd (*n* = 76, estimates with 95% CIs in parentheses).

Metric	MLRM ^1^	SCC1 ^2^	SCC3 ^3^
Accuracy	0.75 (0.64–0.84)	0.65 (0.53–0.75)	0.66 (0.54–0.76)
Balanced Accuracy	0.75 (0.66–0.85)	0.66 (0.56–0.76)	0.66 (0.55–0.76)
Sensitivity	0.80 (0.63–0.92)	0.86 (0.70–0.95)	0.63 (0.45–0.79)
Specificity	0.71 (0.55–0.84)	0.46 (0.31–0.63)	0.68 (0.52–0.82)
Positive predictive value	0.70 (0.54–0.83)	0.58 (0.43–0.71)	0.63 (0.45–0.79)
Negative predictive value	0.81 (0.64–0.92)	0.80 (0.58–0.93)	0.68 (0.52–0.82)
Cohen’s kappa	0.50 (0.31–0.70)	0.31 (0.10–0.52)	0.31 (0.10–0.53)
AUC	0.76 (0.65–0.87)	-	-
Brier Score	0.20 (0.16–0.24)	-	-

^1^ MLRM = multivariable logistic regression model, corresponding to the model that was proposed by this study. ^2^ SCC1: rule-based algorithm adapted from Rowe et al. [19]. Cows were classified as having intramammary infection when Somatic Cell Count was ≥200,000 cells/mL in at least one of the Dairy Herd Improvement tests or when more than one clinical mastitis case was recorded during the current lactation. ^3^ SCC3: rule-based algorithm adapted from Lipkens et al. [29]. Cows were classified as having intramammary infection when Somatic Cell Count was ≥200,000 cells/mL in at least one of the last three Dairy Herd Improvement tests before dry-off.

**Table 7:** Pairwise comparison of classification performance between the proposed model and the two rule-based algorithms on the validation set (*n* = 76), assessed with McNemar’s exact test.

Comparison ^1^	Correct Only with A, *n*	Correct Only with B, *n*	Discordant Pairs, *n*	*p*	Holm Adjusted *p*
SCC1 vs. SCC3	8	9	17	1.000	1.000
SCC1 vs. MLRM	6	14	20	0.115	0.345
SCC3 vs. MLRM	6	13	19	0.167	0.345

^1^ All three models were evaluated on the same validation cows, so comparisons are paired. Discordant pairs are cows classified correctly by one model and incorrectly by the other; concordant pairs carry no information for the test. *p*-values are from McNemar’s exact test, adjusted for multiple comparisons with the Holm method. MLRM = multivariable logistic regression model, corresponding to the model that was proposed by this study. SCC1: rule-based algorithm adapted from Rowe et al. [19]. Cows were classified as having intramammary infection when Somatic Cell Count was ≥200,000 cells/mL in at least one of the Dairy Herd Improvement tests or when more than one clinical mastitis case was recorded during the current lactation. SCC3: rule-based algorithm adapted from Lipkens et al. [29]. Cows were classified as having intramammary infection when Somatic Cell Count was ≥200,000 cells/mL in at least one of the last three Dairy Herd Improvement tests before dry-off.

## Data Availability

The data presented in this study are available on request from the corresponding author.

## Abbreviations

The following abbreviations are used in this manuscript:

BDCT	Blanket Dry Cow Therapy
IMI	Intramammary Infection
SDCT	Selective Dry Cow Therapy
SCC	Somatic Cell Count
DHI	Dairy Herd Improvement
SSLO	Streptococcus and streptococcus-like organisms
gen_cell	Genetic value for Somatic Cell Count
LS	Linear Score
MLRM	Multivariable Logistic Regression Model
VIF	Variance Inflation Factors

## Appendix A

**Table A1:** Descriptive statistics of the continuous candidate predictors, overall and stratified by intramammary infection (IMI) status at dry-off.

Variable ^1^	Overall (*n* = 301)			No IMI (*n* = 172)			IMI (*n* = 129)		
	Mean (SD)	Median (Min–Max)	95% CI	Mean (SD)	Median (Min–Max)	95% CI	Mean (SD)	Median (Min–Max)	95% CI
LS_L1 ^†^	3.62 (1.49)	3.60 (0.10–8.00)	3.45–3.79	3.17 (1.38)	3.10 (0.10–7.10)	2.96–3.37	4.22 (1.41)	4.10 (0.60–8.00)	3.98–4.47
LS_L2	3.51 (1.49)	3.40 (0.10–8.70)	3.34–3.68	3.05 (1.37)	3.00 (0.10–7.60)	2.84–3.26	4.12 (1.43)	4.10 (0.60–8.70)	3.87–4.37
LS_L3 ^†^	3.43 (1.54)	3.20 (0.10–7.80)	3.25–3.60	2.93 (1.36)	2.80 (0.10–7.80)	2.72–3.13	4.09 (1.52)	4.00 (1.50–7.70)	3.83–4.35
LS_L4	3.37 (1.58)	3.20 (0.00–9.20)	3.19–3.55	2.90 (1.51)	2.70 (0.00–8.20)	2.67–3.13	4.00 (1.45)	3.80 (1.00–9.20)	3.74–4.25
LS_L5	3.13 (1.61)	3.00 (0.10–7.40)	2.95–3.31	2.66 (1.52)	2.40 (0.10–7.40)	2.43–2.89	3.76 (1.51)	3.70 (0.50–7.20)	3.49–4.02
LS_L6	3.03 (1.68)	2.80 (0.10–7.80)	2.84–3.23	2.62 (1.63)	2.40 (0.10–7.50)	2.38–2.87	3.59 (1.60)	3.40 (0.10–7.80)	3.31–3.87
milk_dry-off (kg)	12.52 (4.35)	12.0 (1.0–25.0)	12.02–13.01	12.79 (4.73)	13.0 (1.0–25.0)	12.08–13.50	12.16 (3.79)	12.0 (2.0–23.0)	11.50–12.81
gen_cell ^†^	100.49 (4.29)	100.0 (88.0–115.0)	100.00–100.97	101.53 (4.28)	101.0 (90.0–115.0)	100.89–102.18	99.09 (3.90)	99.0 (88.0–108.0)	98.41–99.77

^1^ IMI was defined by dry-off culture as at least one quarter positive for a major pathogen. LS_L1-LS_L6 denote the linear score of the last six test-days before dry-off, indexed by proximity to dry-off (LS_L1 = last test); all cows had at least six test-day records, so no values were missing. milk_dry-off = milk yield at dry-off (kg); gen_cell = genetic index for somatic cell count (values above 100 indicate greater genetic resistance). SD = standard deviation; CI = confidence interval. ^†^ Predictor retained in the final multivariable model.

**Table A2:** Distribution of the categorical candidate predictors, overall and stratified by intramammary infection (IMI) status at dry-off.

Variable ^1^	Category	Overall (*n* = 301)		No IMI (*n* = 172)		IMI (*n* = 129)	
		*n* (%)	95% CI	*n* (%)	95% CI	*n* (%)	95% CI
num_mast ^†^	≥1 clinical mastitis case	61 (20.3%)	16.1–25.2	25 (14.5%)	10.0–20.6	36 (27.9%)	20.9–36.2
teat_level	(≥1 teat with score >2)	73 (24.3%)	19.8–29.4	48 (27.9%)	21.7–35.0	25 (19.4%)	13.5–27.0
lact	1	110 (36.5%)	31.3–42.1	77 (44.8%)	37.5–52.2	33 (25.6%)	18.8–33.7
lact	2	87 (28.9%)	24.1–34.3	46 (26.7%)	20.7–33.8	41 (31.8%)	24.4–40.2
lact	>2	104 (34.6%)	29.4–40.1	49 (28.5%)	22.3–35.6	55 (42.6%)	34.4–51.3

^1^ Values are counts and percentages within each column, with 95% Wilson confidence intervals. CI = confidence interval. num_mast = clinical mastitis recorded during the current lactation, dichotomised as at least one case versus none; teat_level = altered teat-end condition, defined as at least one teat with a score above 2; lact = parity class (first lactation, second lactation, greater than second lactation). Binary predictors are presented as a single row reporting the count in the positive category, the complementary category being its exact complement. Parity is presented as three rows, one per class, since the variable has three levels; in the model it was coded as two dummy variables, with the first-lactation class as the reference category. Data were complete for all 301 cows. No hypothesis tests are reported for these descriptive comparisons. ^†^ Predictor retained in the final multivariable model.

**Table A3:** Confusion matrix on the validation set (*n* = 76) for the proposed multivariable logistic regression model (MLRM) classifying major-pathogen intramammary infection at dry-off.

Model Classification ^1^	Major-Pathogen IMI	No Major-Pathogen IMI	Total
Classified as infected	28	12	40
Classified as non-infected	7	29	36
Total	35	41	76

^1^ Classification based on the probability threshold of 0.35 selected on the training set. Infection status was defined by dry-off culture as at least one quarter positive for a major pathogen. The 7 cows in the lower-left cell are cows infected by major pathogens that would have been left untreated, and the 12 in the upper-right are cows without a major-pathogen infection that would have been treated unnecessarily.
